# An Introduction to Applied Bioinformatics: a free, open, and interactive text.

**DOI:** 10.21105/jose.00027

**Published:** 2018-10-02

**Authors:** Evan Bolyen, Jai Ram Rideout, John Chase, T. Anders Pitman, Arron Shiffer, Willow Mercurio, Matthew R Dillon, J Gregory Caporaso

**Affiliations:** 1 Pathogen and Microbiome Institute, Northern Arizona University, Flagstaff, AZ, USA.; 2 Department of Biological Sciences, Northern Arizona University, Flagstaff, AZ, USA.

## Summary

### Statement of need:

Due to the increasing rate of biological data generation, bioinformatics is rapidly growing as a field and is now an essential part of scientific advances in human health and environmental sciences. Online and publicly accessible resources for learning bioinformatics exist (e.g., Rosalind, (Searls, 2012, 2014)), and there are excellent textbooks and courses in the area, some focused heavily on theory (Durbin, Eddy, Krogh, & Mitchison, 1998; Felsenstein, 2003), and others geared toward learning specific skills such as Python programming or the Unix shell (Dunn & Haddock, 2010; Wilson, 2016). An Introduction to Applied Bioinformatics (IAB) is a free, online bioinformatics text that bridges the gap between theory and application by teaching fundamentals of bioinformatics in the context of their implementation, using an interactive framework based on highly relevant tools including Python 3, Jupyter Notebooks, and GitHub.

IAB is geared toward students who are completely new to bioinformatics, though having completed an introductory course (or book) in both Computer Science and Biology are useful prerequisites. IAB readers begin on the project website. While it is possible to view the content statically from this page, we recommend that readers work interactively by installing IAB. Readers progress through chapters that introduce fundamental topics, such as sequence homology searching and multiple sequence alignment, and presents their Python 3 implementation. Because the content is presented in Jupyter Notebooks, students can edit and execute the code, for example to explore how changing k-word size or an alignment gap penalty might impact the results of a database search. The Python code that readers interact with is intended for educational purposes, where the implementation is made as simple as possible, sometimes at the cost of computational efficiency. Chapters therefore also include examples of performing the same analyses with scikit-bio, a production-quality bioinformatics Python 3 library. This enables a rapid transition from learning theory, or how an algorithm works, to applying techniques in a real-world setting.

IAB additionally contains Wikipedia-style “Edit” links in each section of the text. When one of these links is followed, the reader is taken to the GitHub online editor where they can submit a pull request to modify content or code. Readers are therefore introduced to GitHub through a user-friendly web interface, and can begin building their GitHub activity history (commonly reviewed by bioinformatics hiring managers). Finally, every time a change is proposed via GitHub, all of the executable content of IAB is automatically tested. This continuous integration testing ensures that IAB example code remains functional as changes are introduced, solving an issue that plagues printed applied computational texts (for example because they describe an outdated software interface).

IAB evolved from lecture materials developed by Dr. Caporaso for an introductory bioinformatics course targeted toward computer science and biology undergraduates (typically juniors or seniors) at Northern Arizona University. Since the early stages of its development, it has been used to teach at least ten courses and short (e.g., one day) bioinformatics workshops. As it became clear that the content and format was useful for teaching bioinformatics, Dr. Caporaso applied for and received grants from the Arizona Technology and Research Initiative and the Alfred P Sloan Foundation to further develop the resource.The content was originally written in Jupyter Notebooks, but as the project grew, it became difficult to maintain the notebooks and in particular to review submissions from others. The Jupyter Notebooks were transitioned to markdown files which are now the source for static HTML and Jupyter Notebook renderings of the content.

The current version of IAB contains six chapters covering fundamental concepts and their applications. It is a dynamic resource that will be expanded, revised and updated over time. Its lifecycle is thus more similar to an active software project than a textbook: a practical approach to education in a rapidly changing field.
